# Medial approach to treat humeral mid-shaft fractures: a retrospective study

**DOI:** 10.1186/s13018-016-0366-1

**Published:** 2016-03-17

**Authors:** Shun Lu, Junwei Wu, Shihong Xu, Baisheng Fu, Jinlei Dong, Yongliang Yang, Guodong Wang, Maoyuan Xin, Qinghu Li, Tong-Chuan He, Fu Wang, Dongsheng Zhou

**Affiliations:** Department of Orthopedics, Shandong Provincial Hospital Affiliated to Shandong University, Jinan, Shandong 250021 China; Department of Orthopaedic Surgery and Rehabilitation Medicine, The University of Chicago Medical Center, Chicago, IL 60637 USA

**Keywords:** Humeral mid-shaft fractures, Medial approach, Plate fixation

## Abstract

**Background:**

Plate fixation is the gold standard for diaphyseal fracture management, and the anterolateral approach is widely used by reconstructive surgeons. However, the outcomes of humeral shaft fracture fixation using a medial approach are rarely reported. The aim of this study is to explore the management and outcomes of humeral mid-shaft fractures fixed through a medial incision.

**Methods:**

Thirty-four patients who sustained a humeral mid-shaft fracture and underwent an open-reduction internal fixation (ORIF) in our department between January 2010 and January 2013 were included in this study. Sixteen patients had an ORIF performed through a medial approach, while the remaining 18 were fixed through an anterolateral approach. Postoperative clinical and radiographic results were reviewed.

**Results:**

There were no significant differences in the blood loss and the range of motion of the shoulder and elbow between the anterolateral and medial fixation groups. One patient in the medial group and two patients in the anterolateral group had radial nerve dysfunction that improved after 8, 3 and 6 weeks, respectively. All patients healed radiographically except one from the anterolateral group who underwent grafting and re-fixation for a non-union. No vascular injuries, infections, malunions, broken plates or loose screws were noted in either group.

**Conclusions:**

The medial approach to the humerus had equivalent outcomes to anterolateral fixation. It is an available choice for humeral mid-shaft fracture fixation in cases where there is no need to expose the radial nerve. The medial approach does not require a pre-bent plate and creates a large operative exposure. A well-hidden incision can also be designed, improving cosmetic outcomes. However, the medial approach is not suitable to proximal or distal humerus fractures.

## Background

Plate fixation is the gold standard for the surgical management of humeral mid-shaft fractures [1–3], and the anterolateral approach is most commonly used [4–7]. However, the medial approach is rarely discussed for humeral shaft fracture management [8]. This is because of the complicated anatomy of the medial aspect of the upper arm.

We believe that there are several merits to the medial approach for humeral mid-shaft fracture management, such as no need to expose the radial nerve and no need to pre-bent the plate and a well-hidden incision. So the aim of this study is to explore the management and outcomes of humeral mid-shaft fracture fixation with a medial approach and evaluate the safety, efficacy and benefits of this approach.

## Methods

Permission for this retrospective study was obtained from the medical ethics committee of Shandong Provincial Hospital Affiliated to Shandong University.

A total of 34 patients who sustained a humeral mid-shaft fracture and underwent an open-reduction and internal fixation (ORIF) at our department between January 2010 and January 2013 were included in this study. There were 22 males and 12 females with ages ranging from 18 to 59 years. The initial injuries were traffic accidents in 19 cases, falls in nine cases and sport-related injuries in six cases. According to the AO/Orthopaedic Trauma Association classification, 18 fractures were type A, nine were type B and seven were type C fractures (Table 1).

**Table 1:** The Characteristics of patients between the two groups

Characteristics	Medial incision group	Anterolateral incision group
Number of cases	16	18
Gender (male/female)	10/5	12/7
Mean age, years (range)	33.6 (18–56)	35.2 (23–59)
Standard deviation (SD)	8.6	6.9
Mechanism of injury		
Traffic accident	9	10
Fall	5	4
Sport injury	2	4
Fracture type (AO/OTA Classification)		
Type A	8	10
Type B	5	4
Type C	3	4

The medial approach was used on 16 patients, while 18 patients underwent anterolateral fixation. All procedures were performed by a senior trauma surgical team. Those injuries associated with an ipsilateral upper limb fracture or a neurovascular injury were excluded from this study.

Data on clinical outcomes, operative time and operative complications were collected and reviewed. Postoperatively, patients were assessed radiographically 1, 2, 3, 6, 9 and 12 months after surgery, with annual imaging thereafter. The range of motion (ROM) of the shoulder and elbow joints was also assessed.

### Surgical technique

#### Medial approach

**Fig. 1:**
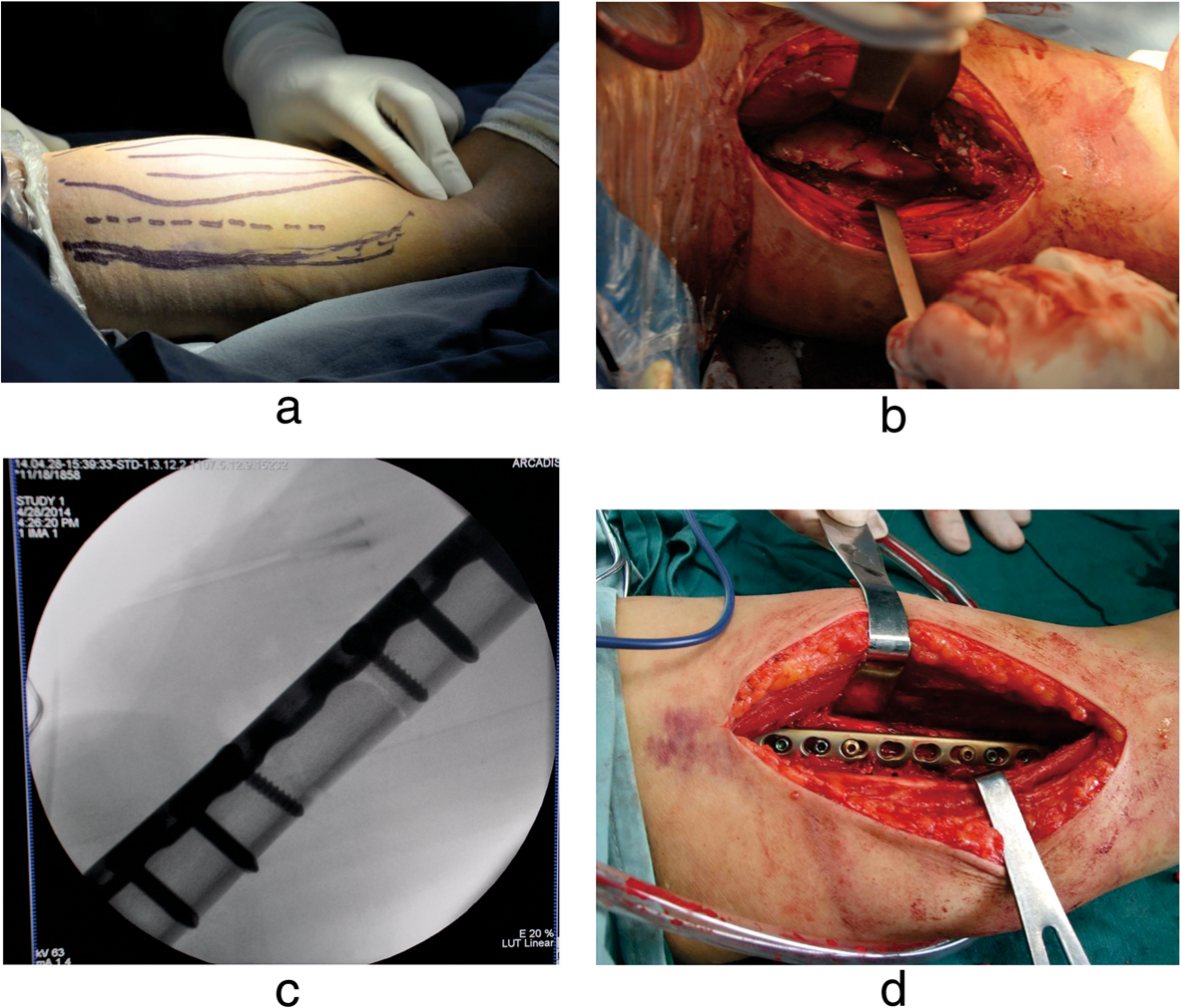
**a** Mark the incision before operation. **b** Expose the fracture region. **c** Fluoroscopy after fixation. **d** Place the plate and fixation

Patients were placed in the supine position with their injured limb in 90° of abduction. A medial incision was created over the fracture site along a line connecting the armpit with the medial condyle of the humerus. The median nerve and the brachial vessels were dissected. Unlike the radial nerve, which is directly attached to the humerus, in the medial approach, the ulnar nerve runs in a relatively superficial location and was therefore easy to dissect. The ulnar nerve also had a high degree of relaxation and was easy to retract away from the fracture site to create ample operative space. The biceps and the triceps were retracted with the neurovascular structures, allowing the triceps to protect the ulnar nerve and brachial artery. The brachialis was longitudinally incised to create a clear fracture exposure. The plate was placed on the anteromedial aspect of the humerus after a satisfactory reduction was performed. X-ray imaging was used to verify proper plate placement (Fig. 1).

#### Anterolateral approach

We performed plate fixation through the anterolateral incision using a previously described technique [4–7]. However, the exploration and protection of the radial nerve through this approach is routinely performed in our department.

### Statistical analysis

All results were evaluated using SPSS 19.0 for Windows (IBM, Chicago, IL, USA). Differences in patient characteristics between the medial and anterolateral approaches such as sex, mechanism of injury and fracture classification were compared along with clinical and radiographic outcomes using a chi-squared test. Patient age and blood loss were compared using Student’s *t* test. Complications including the rates of nerve injury and fracture non-union were assessed using Fisher’s exact test. In all cases, statistical significance was defined as *p* < 0.05.

## Results

The average age of patients who underwent a medial approach was 33.6 years (range 18 to 56 years), with a male/female ratio of 2:1. The average age of patients who underwent an anterolateral approach was 35.2 years (range 23 to 59 years), with a similar male/female ratio to the medial group. There were no significant differences between the average age, gender composition, mechanism of injury and fracture type between the two surgical approach groups.

Blood loss was 271.88 ± 61.23 ml in the medial group and 278.33 ± 93.29 ml in the anterolateral group. There was no significant difference in blood loss between the approaches (*p* > 0.05) (Table 2).

**Table 2:** The Blood loss and Complications between the two groups

Operative records	Medial incision group	Anterolateral incision group	*t*/*χ*2 value	*p* value
Blood loss (ml)	271.875 ± 61.234	278.333 ± 93.290	0.815	–
Complications				
Nerve disturbances	1	2	–	1.000
Non-union	0	1	–	1.000

Radiographic healing was observed 3 months after surgery in 14 of 16 patients in the medial approach group. The remaining two patients healed after a longer follow-up period. Radiographic healing was observed in 15 of 18 patients 3 months following surgery. Two patients healed after a longer follow-up. One patient required iliac bone grafting for a non-union 1 year after surgery and went on to good healing.

**Fig. 2:**
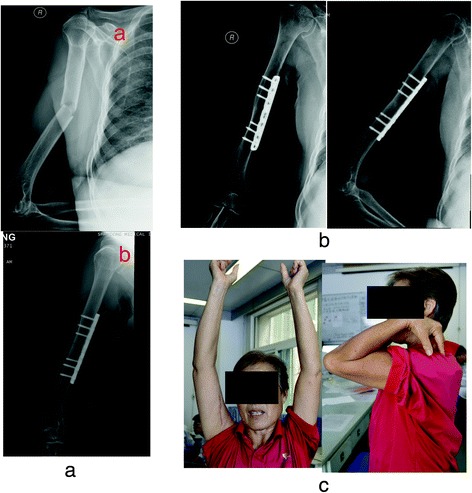
Female, 56 years, who was fall from a height, the fracture type: A, and she was taken ORIF by medial incision. **a** *a* The X-ray after injury and *b* the X-ray after ORIF. **b** The X-ray at 1 year after operation. **c** The ROM of shoulder and elbow joint

Full range of motion (ROM) of the shoulder was restored in both groups postoperatively. All patients in the medial group had full ROM of their elbow joint without postoperative restriction. Two patients in the anterolateral group had slightly reduced elbow extension in the early postoperative period, although full elbow ROM was restored 1 to 2 months later (Fig. 2).

One patient in the medial approach group developed a radial nerve palsy after surgery. This may be owing to operative manipulation. Normal function returned 8 weeks after surgery. Two radial nerve palsies occurred in the anterolateral approach group, with a recovery in both patients following the use of neurotrophic drugs for 3 and 6 weeks after surgery. One patient in the anterolateral group developed a non-union after the ORIF, requiring re-fixation with autologous iliac bone graft using the same surgical approach to achieve a union. All patients in the medial approach group achieved a union after the operation. No vascular injury, infection, fracture displacement, plate fracture, screw extrusion, plate breakage or screw loosening was observed in either group.

## Discussion

Plate fixation is considered the gold standard for humeral mid-shaft fracture fixation [1–3]. The anterolateral approach is widely accepted for the treatment of these injuries [4–7]. The medial approach is another choice for humeral shaft fracture fixation but has been rarely discussed. The medial approach was first reported by Judet in 1968 [8]. Jupiter [9] later reported that it could be used in cases of complex non-unions of the humeral diaphysis. The main reason that the medial approach is not widely used may be the complicated anatomy of the medial aspect of the upper arm. Surgeons tend to choose an approach with fewer nerves and blood vessels, and the brachial vessels, median nerve and ulnar nerve are visible during a medial approach.

The anterolateral approach is widely accepted for mid-shaft humeral fracture fixation [4–7]. However, in our evaluation of the medial approach, we found that there are no significant differences in the blood loss, fracture healing rate and postoperative function of patients treated with either approach. Moreover, there are several merits to the medial approach. We therefore have adopted the medial approach for diaphyseal humeral fractures at our institution.

### Operative site exposure

**Fig. 3:**
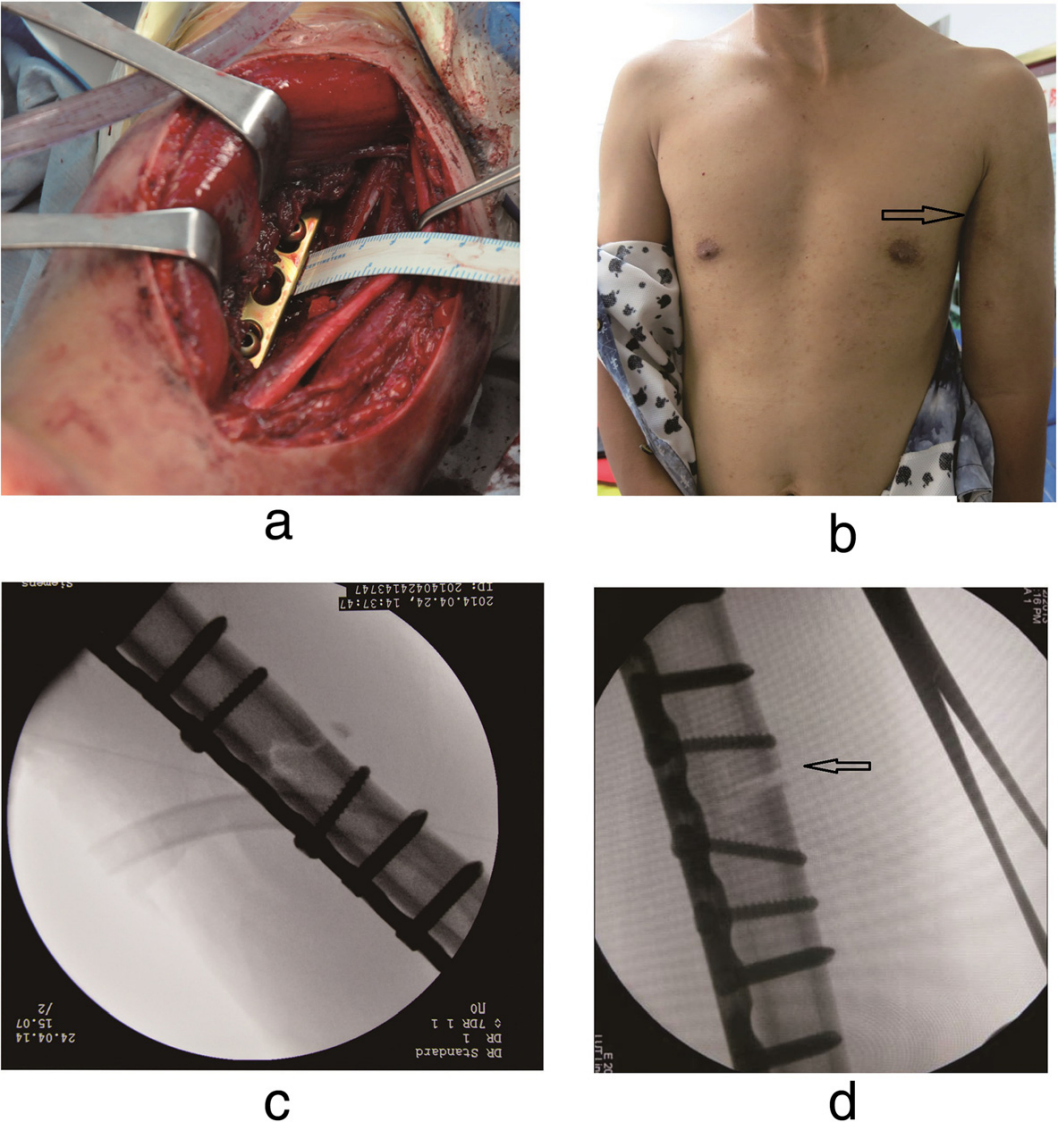
**a** The ulnar nerve is easy to pull away from the fracture site with at least 3.5 cm. **b** In the medial approach, the incision was secluded. **c** In the medial approach, the plate could be placed directly without being reshaped. **d** In the anterolateral approach, gap widen (indicated by the *arrows*) often occurs in the medial of fracture site when the fixation is undertaken

The anterolateral approach is the classic approach for humeral shaft fracture fixation. It allows for excellent fracture exposure and fixation. In the medial approach, the brachial vessels and the median nerve run in a relatively superficial location, making them easy to explore. It is also possible to create a clear exposure by splitting the brachialis after properly protecting the vessels and nerves. We found that the ulnar nerve is relaxed enough that it can be easily pulled away at least 3.5 cm from the fracture site (Fig. 3a), ensuring an ample operative space for reduction and fixation. In contrast, in the anterolateral approach, the radial nerve is strained and requires meticulous protection to reduce the incidence of nerve damage. Reports of radial nerve injury are common [9–12].

The biggest limitation of the medial approach is the reduced ability for the surgeon to extend the exposure compared to the anterolateral approach. It is therefore difficult to expose and manipulate both the proximal and distal humerus through the medial approach. We therefore only recommend the medial approach for mid-shaft fractures without proximal or distal humerus involvement.

### Nerve exploration and damage

It is controversial whether or not the radial nerve needs to be exposed in humeral shaft fractures without clinical evidence of nerve palsy. It is reported that more than 80 % of radial nerve injuries recover spontaneously [10, 13], and early detection is advocated to reduce iatrogenic radial nerve injuries. However, despite radial nerve exploration, intraoperative traction, direct nerve contact with the plate, nerve compression by scar tissue and bony callus are still risk factors for radial nerve injury. In our study, there were two radial nerve palsies after fixation using the anterolateral approach that improved after conservative treatment with neurotrophic drugs for 3 and 6 weeks. No long-term radial nerve paralysis occurred.

Among those who were fixed medially, one patient developed a radial nerve palsy that resolved in 8 weeks. This may be owing to operative manipulation. No ulnar nerve or other neurovascular injury occurred. We believe that the neurovascular structures on the medial arm are superficial and easily detected during the medial approach, and an ample operative space can be created because of the high degree of relaxation of the ulnar nerve. Compared with the anterolateral approach, it may also be possible to get better soft tissue coverage over the plate using the medial approach.

### Plate placement

The cross-sectional shape of the humerus from the mid-shaft to the distal metaphysis is triangular. It has three aspects: anteromedial, anterolateral and posterior [14, 15]. In the anterolateral approach, the lateral aspect of the humerus is uneven, often leading to medial gapping during fixation (Fig. 3c, d). The plate often needs to be pre-bent during placement. During the medial approach, the plate can be placed without being reshaped onto the smooth anteromedial humerus. Biomechanically, the plate should be placed on the tension side of the injury [16]. The implant should therefore be placed on either the anterolateral or the posterior aspects of the bone. Unlike the femur or tibia, whose primary stresses are weight-bearing, the major stresses on the humerus are rotational forces. The plate can therefore be placed on the medial aspect of the humerus [17].

### The scars of the skin

In the medial approach, the incision is partially hidden, which may result in an improved long-term cosmesis that is particularly beneficial for patients with particular cosmetic demands (Fig. 3d). Scars are difficult to find after surgery and can therefore meet patients’ aesthetic requirements.

### Plate removal

In some cases, patients require plate removal. In these cases, the direct contact of the radial nerve with the plate and the increased local scar tissue and bony callus results in increased rates of radial nerve injuries. Running superficial to the radial nerve, the neurovascular structures of the medial arm can be explored easily. There were two patients at our institution who underwent plate removal, and no nerve injury resulted.

**Fig. 4:**
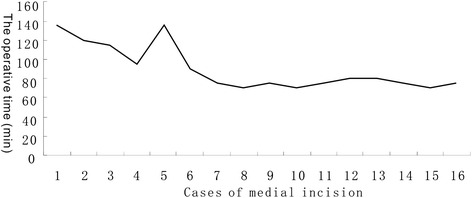
The learning curve of applied ORIF through medial approach

The manipulation necessary to perform a medial approach requires a significant learning curve. Familiarity with the local anatomy is needed to avoid neurovascular injury. The time to complete the ORIF through a medial approach was recorded, and the learning curve of each surgeon was plotted (Fig. 4). In early cases, the ORIF took approximately 120 min. This time was decreased significantly in subsequent cases to a mean operative time of 90 min.

As the brachial vessels, median nerve and ulnar nerve go through the medial aspect of the arm, surgeons tend to choose other approaches that manipulate fewer critical structures. However, we were able to create a clear exposure in our study. Neurovascular injury can be avoided through meticulous manipulation and cautious neurovascular protection. We therefore believe that a complex anatomy should not be the reason for avoiding a medial approach.

When managing humeral mid-shaft fractures through the medial approach, there is no need to expose the radial nerve and no need to pre-bend the plate before fixation. An ample operative space could be created through a cosmetically occult incision. We therefore recommend that a medial approach be used in the following cases: (1) humeral mid-shaft fractures without a radial nerve injury; (2) humeral shaft fractures associated with a medial neurovascular injury that requires surgical exploration and repair; (3) a laceration or soft tissue disruption on the lateral arm and (4) patients with significant cosmetic requirements. Patients with distal and proximal humeral fractures or radial nerve injuries are not indicated for this approach.

## Conclusions

The medial approach to the humerus is a possible choice for humeral mid-shaft fractures. However, humeral shaft fractures with radial nerve damage require nerve exploration, and a medial approach should not be performed. As this study was based on a small sample size and a retrospectively analysis of early results and short-term complications, a larger sample size and a longer follow-up period are required to fully study this approach.

### Consent statement

Written informed consent was obtained from the patients for publication of this report and accompanying images. A copy of the written consent is available for review by the Editor-in-Chief of this journal.
